# Evidence of thermophilisation and elevation-dependent warming during the Last Interglacial in the Italian Alps

**DOI:** 10.1038/s41598-018-21027-3

**Published:** 2018-02-08

**Authors:** V. E. Johnston, A. Borsato, S. Frisia, C. Spötl, Y. Dublyansky, P. Töchterle, J. C. Hellstrom, P. Bajo, R. L. Edwards, H. Cheng

**Affiliations:** 1 Museo delle Scienze, Corso del Lavoro e della Scienza, 3, 38122 Trento, Italy; 2 School of Environmental and Life Sciences, University of Newcastle, Callaghan, 2308 NSW Australia; 3 Institute of Geology, University of Innsbruck, Innrain 52, 6020 Innsbruck, Austria; 4 School of Earth Sciences, University of Melbourne, Melbourne, 3010 VIC Australia; 5 Department of Earth Sciences, University of Minnesota, Minneapolis, USA; 6 Institute of Global Environmental Change, Xi’an Jiaotong University, Xi’an, China; 7 Present Address: Karst Research Institute, Research Centre of the Slovenian Academy of Sciences and Arts, Titov trg 2, SI-6230 Postojna, Slovenia

## Abstract

Thermophilisation is the response of plants communities in mountainous areas to increasing temperatures, causing an upward migration of warm-adapted (thermophilic) species and consequently, the timberline. This greening, associated with warming, causes enhanced evapotranspiration that leads to intensification of the hydrological cycle, which is recorded by hydroclimate-sensitive archives, such as stalagmites and flowstones formed in caves. Understanding how hydroclimate manifests at high altitudes is important for predicting future water resources of many regions of Europe that rely on glaciers and snow accumulation. Using proxy data from three coeval speleothems (stalagmites and flowstone) from the Italian Alps, we reconstructed both the ecosystem and hydrological setting during the Last Interglacial (LIG); a warm period that may provide an analogue to a near-future climate scenario. Our speleothem proxy data, including calcite fabrics and the stable isotopes of calcite and fluid inclusions, indicate a +4.3 ± 1.6 °C temperature anomaly at ~2000 m a.s.l. for the peak LIG, with respect to present-day values (1961–1990). This anomaly is significantly higher than any low-altitude reconstructions for the LIG in Europe, implying elevation-dependent warming during the LIG. The enhanced warming at high altitudes must be accounted for when considering future climate adaption strategies in sensitive mountainous regions.

## Introduction

Interglacials are warm periods in the Quaternary, where analogues to present and future warming trends can be sought. The Last Interglacial period (LIG; ~130–115 ka) is considered a partial analogue for a ~2 °C global warming scenario, with ice-volume loss and higher sea-levels^1^. How this translated into regional to local-scale climate and ecosystem responses, to test downscaling and probability models and devise adaptation strategies, is still poorly known for most regions. This is feasible only through a high-density network of climate proxy archives, particularly covering sensitive mountain regions that hold central Europe’s principle water resources.

Predicting possible trends of climate and vegetation changes for the Alpine region of Europe is particularly challenging because of complex orographic effects on atmospheric circulation that act as a divide of meridional moisture transport. Crucially, water resources necessary to sustain agriculture, tourism and the current standard of living, are present at high altitudes in the Alps as snow, ice and in lakes, both natural and artificial. Alterations in the hydrological cycle associated with climate change could potentially affect annual and seasonal discharge of major rivers that originate in the Alps^2^. Furthermore, thermophilisation associated with climate warming shifts plant communities upward, limiting ecologic niches for high-alpine species, thus, resulting in conspicuous biodiversity loss^3,4^. Knowledge of past hydrological and ecosystem responses to warming at high altitudes, which echoes high latitudes, is required to design appropriate climate adaptation and conservation strategies. This can be accomplished by extracting proxy data from archives of past interglacials that were warmer than the late Holocene and, specifically, the Anthropocene.

Speleothems, defined as secondary mineral deposits formed in caves^5^, identify one of the most accurate, precisely datable, continental archives of climate and environmental proxy data. Their formation, morphology, mineralogy, structure and chemical composition depend on the cave setting, ventilation dynamics and on the type of vegetation above the cave^6–8^. In the European Alps, speleothem formation is mostly limited to caves located below the timberline, where efficient soil turnover in mixed conifer–deciduous forests promotes elevated biogenic pCO_2_ that combines with rainwater to produce weak carbonic acid that dissolves the carbonate host rock^8^. Subsequent re-deposition of calcite is promoted by degassing of the dripwaters in the cave atmosphere, whereby the critical threshold in the calcite saturation state (SI_CC_) of the film of fluid wetting a speleothem is >0.1 and preferably >0.5 (refs^9,10^). Presence of fossil speleothems in high-altitude caves where significant sparitic calcite deposits are not currently forming, suggests that an upward shift in the timberline, associated with thermophilisation, must have occurred in the past. This implies that warmer than present-day temperatures characterised the region prior to the late Holocene. The impact of a warmer-than-current climate on water resources and vegetation can be elucidated through a multi-proxy record approach reconstructing past climate and environmental processes. Here, we present multi-proxy records from three coeval speleothems that formed during the LIG in a subalpine cave located in the Italian Alps to yield the temperature of the cave catchment area during the LIG. Our reconstructed temperatures are then compared with local- to regional-scale modern and LIG temperature estimates to gain an understanding of ecosystem and hydrological responses to warm interglacial climates in sensitive, mountainous regions.

## Results and Interpretations

### Study site

**Figure 1:**
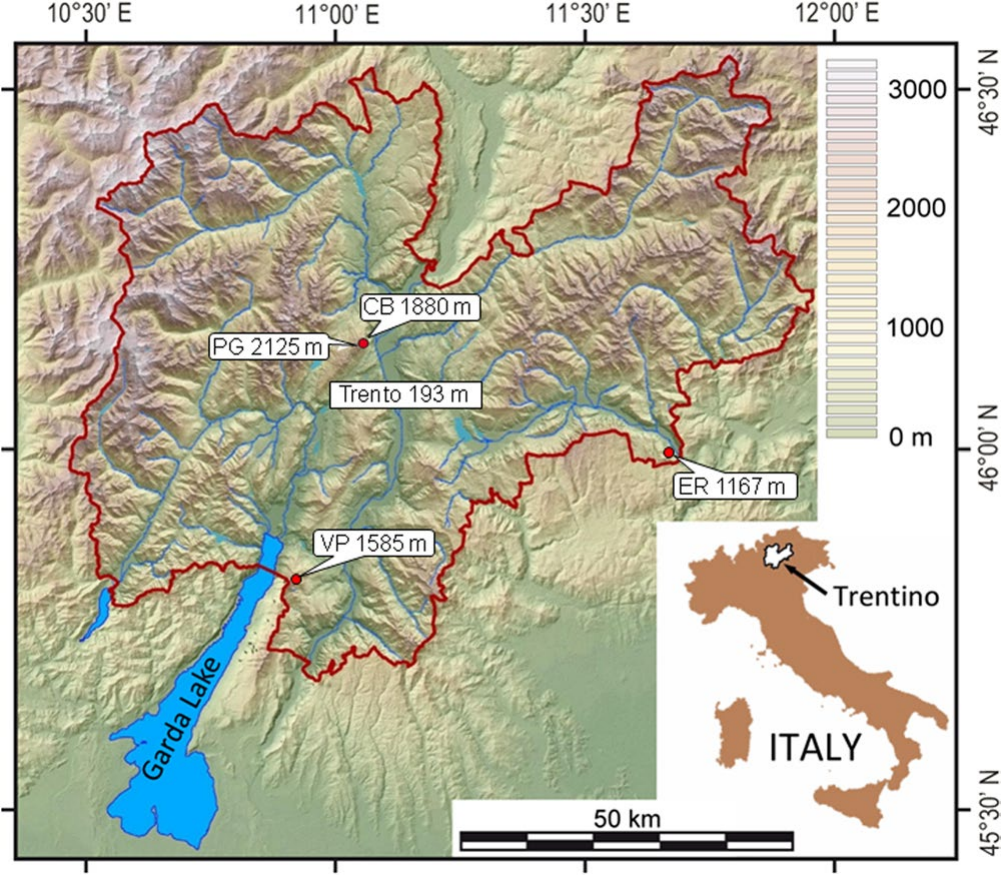
Topographic map of Trentino. The locations of the provincial capital Trento, Mt. Paganella (PG) and the Cesare Battisti (CB) cave are shown alongside their altitudes (m a.s.l.). The locations of the caves Grotta di Ernesto (ER) and Pozzo di Val dal Parol (VP) are also shown, as they provide modern-day analogues for CB Cave during the peak and late LIG, respectively. The maps were generated using the software GRASS GIS version 6.1 (https://grass.osgeo.org/) with topographic data taken from the National Aeronautics and Space Administration’s (NASA) Shuttle Radar Topography Mission (SRTM), available from the U.S. Geological Survey (https://lta.cr.usgs.gov/SRTM).

Cesare Battisti (CB) cave (46°08′N, 11°02′E) opens at 1880 m a.s.l. (metres above sea-level) on a near-vertical, northeast-facing cliff wall of Mt. Paganella, Trentino, Italy (Fig. 1 and Supplementary Fig. S1). The catchment area for cave dripwater is a 0.25 km^2^, gently dipping, northeast-facing plateau reaching 2024 m a.s.l., colonised by dwarf pines (*Pinus mugo*), alpine grasses and shrubs. In pockets of undisturbed soil, the mean annual soil CO_2_ concentration is 3052 ± 2316 ppmV^8^. The 204 m-deep cave system, developed in well-bedded, Early Jurassic limestone, consists of a maze of strongly ventilated passages (Supplementary Fig. S2). Mean annual cave air CO_2_ concentration is 447 ± 108 ppmV and average interior air temperature is 3.8 ± 0.2 °C^8^. Cave passages are decorated by ancient flowstones and stalagmites, while modern and Holocene speleothems consist of thin calcite crusts^11^ and moonmilk^12^ precipitating from barely saturated dripwaters^9^ (SI_CC_ = −0.07 ± 0.18).

### Specimens

Three inactive speleothems were sampled from two locations in CB Cave, characterised by morphologies and fabrics that stem from diverse discharge and parent-water compositions, ensuring the full spectrum of isotope and trace-element characteristics (from equilibrium to kinetically influenced) is accounted for. A 70 mm-thick flowstone (CB25) and a 185 mm-tall stalagmite (CB47) were sampled from the “Scrigno” chamber, with a ceiling height of 0.6 m, located ~50 m below the surface (1930 m a.s.l.). Flowstone CB39 (33 mm-thick) was sampled in a deeper passage located ~130 m below the surface and ~50 m from one of the many cliff wall entrances (Supplementary Figs S2 and S3). Flowstone CB25 is composed of mm-thick layers of porous, open columnar calcite separated by thin laminae of compact columnar calcite (Supplementary Discussion and Fig. S4). CB25 was sampled close to its presumed discharge point and, given its fabric, we expect formation under relatively high flow rates, with minimum disequilibrium isotope fractionation. The cone-shaped stalagmite CB47 consists of porous, open columnar calcite, suggesting relatively fast and constant drip rates, likely resulting in negligible disequilibrium isotope fractionation during formation. In contrast, the dendritic fabric that characterises its upper 35 mm suggests a variable drip rate regime, at a seasonal or annual scale^7^. In flowstone CB39, the lack of intercrystalline porosity within its compact columnar calcite fabric layered with micrite and elongated columnar calcite with lateral overgrowths suggests increased discharge and inclusion of impurities^10^ (Supplementary Discussion and Fig. S3).

### Chronology

Fifteen uranium-series ages of the three speleothem samples show limited detrital thorium contamination and relatively low U concentrations (54–279 ppb) causing large uncertainties on the corrected ages (0.5–4 ka, at 2 standard errors) (Supplementary Table S5). Open columnar fabrics imply that a small amount of U-leaching could be expected. The age models (Supplementary Fig. S6) indicate that CB25 formed from 125.2 ± 1.4 ka to 121.3 ± 1.0 ka, CB47 from 126.5 ± 1.1 ka to 123.6 ± 1.2 ka and CB39 from 127.8 ± 1.3 ka to 120.3 ± 1.5 ka. These ages place the formation of all three speleothems during the LIG^13–16^.

### Stable isotopes

**Figure 2:**
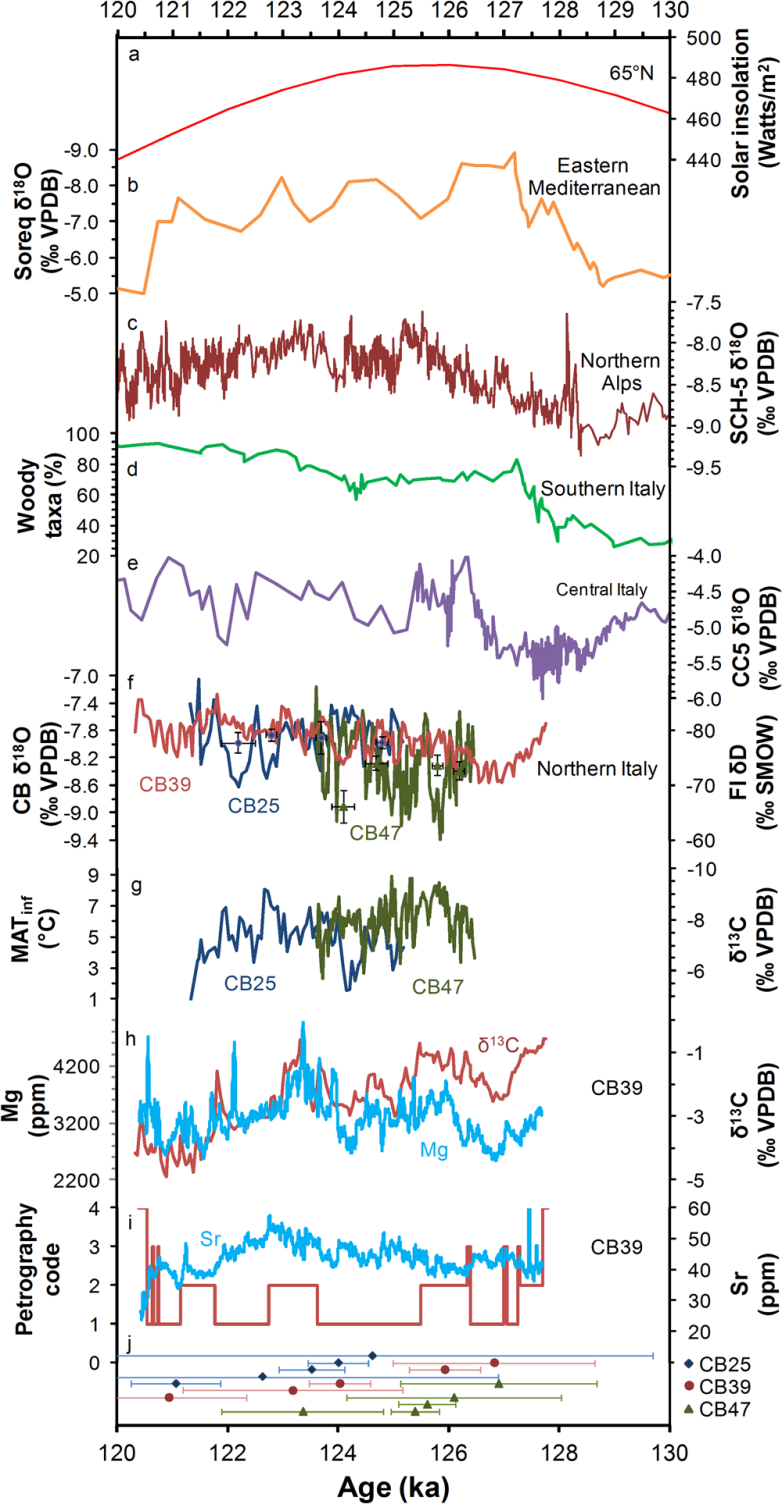
Comparison of CB Cave proxy data with published time series. (**a**) Summer solar insolation at 65°N (ref.^22^), (**b**) composite δ^18^O time series from Soreq Cave, Israel, speleothems^14^, (**c**) δ^18^O time series from stalagmite SCH-5 from Schneckenloch Cave in the Austrian Alps^23^, (**d**) percentage of woody taxa at Lago Grange di Monticchio, southern Italy^21^, (**e**) δ^18^O time series of flowstone CC5 from Corchia Cave, Italy^24^, (**f**) δ^18^O time series of CB Cave speleothems with discrete measurements of FI δD values, (**g**) δ^13^C time series for CB47 and CB25 and the corresponding mean annual temperature of the infiltration area (MAT_inf_), (**h**) δ^13^C values and Mg concentration time series from CB39, (**i**) petrographic code and Sr concentrations of CB39 and (**j**) U-series ages and uncertainties for the CB Cave speleothems. Petrographic code: 1) elongate columnar calcite, 2) elongate columnar calcite with lateral overgrowths, 3) micrite and 4) detritus.

Mean δ^13^C values of flowstone CB39 (−2.4‰ ± 1.1‰) are similar to those of modern calcite formed in the Scrigno chamber (−2.3‰ ± 1.9‰), which are modified by disequilibrium fractionation^11^. δ^13^C values of CB25 and CB47 are significantly lower (<4‰) than modern calcite δ^13^C values, at −7.2‰ ± 0.9‰ (CB25) and −8.1‰ ± 0.8‰ (CB47), in accordance with isotope fractionation closer to equilibrium conditions. δ^18^O values of the three speleothems are comparable: CB47 at −8.3‰ ± 0.5‰, while CB25 and CB39 have identical means of −7.9‰ ± 0.3‰ (Fig. 2f and Supplementary Material S7–S10). These are slightly lower (<1‰) than δ^18^O values of modern calcite collected from the cave (−7.4‰ ± 0.2‰)^11^.

### Speleothem fluid inclusions (FI)

Stable isotope values from tiny amounts of dripwater trapped within the host calcite as fluid inclusions (FI) from the time of speleothem formation (δ^18^O_FI_ and δD_FI_) were analysed to gain insight on past temperatures. Comparing the δ^18^O_FI_ values with those of the host-calcite (δ^18^O_C_) using calcite–water geothermometer equations allows assessment of speleothem formation temperatures (Table 1 and Supplementary Table S11). For this, the δ^18^O_C_ values must be in isotopic equilibrium with the δ^18^O_FI_ of the coevally trapped inclusions^17^. Soaking blocks of CB speleothems in water of different isotopic composition indicated that there was negligible fluid exchange through the interconnected pore space (see Supplementary Discussion), which signifies that FI data accurately reflect the properties of the dripwater.

**Table 1:** Temperatures calculated based on δ^18^O and δD values of calcite (_C_) and fluid inclusions (_FI_).

	Age^a^ (ka)	δ^18^O_C_^a^ (‰ VPDB)	δ^18^O_FI_ (‰ VSMOW)	δD_FI_ (‰ VSMOW)	δ^18^O_FI_^b^ (‰ VSMOW)	Temperature (°C)^c^
CB25-A	124.8 ± 0.1	−8.1 ± 0.2	−11.3 ± 0.3	−77.9 ± 1.0	−11.3 ± 0.6	3.5 ± 1.8
CB25-B	122.2 ± 0.3	−8.3 ± 0.2	−11.1 ± 0.2	−77.8 ± 1.8	−11.3 ± 0.5	4.6 ± 1.0
CB25-C	123.7 ± 0.1	−8.1 ± 0.2	−9.9 ± 0.6	−78.7 ± 3.0	−11.4 ± 0.8	3.0 ± 2.8
CB25-D	122.8 ± 0.1	−8.2 ± 0.2	−10.5 ± 1.1	−79.2 ± 1.1	−11.4 ± 1.4	3.4 ± 4.9
CB47-A	124.1 ± 0.2	−7.9 ± 0.2	−10.7 ± 1.2	−66.2 ± 2.9	−9.9 ± 1.4	8.9 ± 5.7
CB47-B	125.8 ± 0.1	−8.8 ± 0.4	−12.0 ± 0.5	−73.7 ± 1.9	−10.8 ± 0.8	9.0 ± 1.7
CB47-C	124.7 ± 0.2	−8.4 ± 0.3	−10.0 ± 1.0	−74.0 ± 1.3	−10.8 ± 1.2	7.0 ± 4.3
CB47-D	126.2 ± 0.1	−8.2 ± 0.5	−9.0 ± 0.6	−72.7 ± 1.7	−10.6 ± 0.9	6.9 ± 1.9

^a^Calculated as an average of the values encompassed in the distance covered by the larger FI samples.

^b^*δ*^*18*^*O*_*FI*_ value calculated using the *δ*D_*FI*_ and the modern Paganella-MWL relationship.

^c^Cave interior temperature calculated using the equation of Johnston *et al*.^11^;

*T* (°C) = 17.66 × 1000/(1000 Ln(*α*) + 30.16) − 273.15,

where, 1000 Ln(*α*) = 1000 × Ln ((1000 + *δ*^*18*^*O*_*C*_)/(1000 + *δ*^*18*^*O*_*FI*_)).

### Trace elements

Both Mg and Sr concentrations are significantly lower in the CB47 stalagmite than in the CB39 flowstone. CB39 Mg concentrations (mean 3283 ± 418 ppm) exhibit considerable variability, with a maximum at ~124–123 ka. CB39 Sr concentrations (mean 44 ± 7 ppm) rise rather steadily until reaching a maximum at ~123 ka. Average Mg and Sr concentrations in a representative sector of CB47 stalagmite are 856 ± 171 ppm and 28 ± 5 ppm, respectively.

## Discussion

In caves formed below a well-developed soil cover, often the case for low-altitude sites in temperate climate settings, CO_2_ is produced constantly in the soil, hence, it not a limiting factor for carbonate dissolution. However, the strongly changeable water availability found in such regions thus contributes the environmental variable that dominates carbonate geochemistry. Consequently, rainfall amount is often a key parameter encoded in low-altitude speleothem proxy data^16,18,19^. By contrast, at high-altitude sites, speleothem formation is limited by the soil efficiency and climate parameters that influence soil CO_2_ production. Soil efficiency is a crucial factor that gives rise to adequate CO_2_ production, resulting in an acidic solution capable of dissolving enough host rock to overcome the threshold in SI_CC_ required to precipitate speleothem carbonate once the infiltration waters reach the cave atmosphere. This SI_CC_ threshold typically corresponds to a local altitudinal (or latitudinal) band that coincides with the *speleothem limit*^9^, which defines a change from a warm and highly vegetated speleothem-forming environment to more hostile conditions where sparitic speleothem development is unlikely. Therefore, the presence of sparitic speleothems, which were fed by waters discharged from pure carbonate host rocks, where present dripwater SI_CC_ values lie below zero (e.g., at high-altitude or high-latitude sites), likely identifies periods in the past when higher temperatures enhanced soil productivity and dripwater SI_CC_ above the threshold required for sparitic speleothem formation. Enhanced soil productivity is thought to be associated with thermophilisation and high-altitude greening, which in turn, favours sparitic speleothem development. Below, we investigate whether the LIG speleothem formation in CB Cave was prompted by warmer temperatures than modern and Holocene conditions.

Geochemical data extracted from speleothems are widely used to reconstruct past climate and environmental conditions. δ^13^C values of speleothem calcite generally reflect soil efficiency and vegetation type and density above the cave. The δ^13^C values of CB25 and CB47 are significantly lower than modern values, suggesting enhanced soil microbial activity and root respiration at the time of their formation with respect to current conditions, arguing for significant LIG greening above the cave. This is consistent with the notion that speleothem formation itself required that infiltration waters had higher SI_CC_ than today, as a result of an upward shift of the *sparitic speleothem limit*. It is, therefore, reasonable to infer an upward shift of the LIG timberline to an altitude well above the elevation of the cave’s catchment area.

CB39 δ^13^C values are considerably higher than those of CB25 and CB47. Significantly higher Mg and Sr concentrations in CB39 than in CB47 suggest enhanced water–rock interactions (WRIs) along the longer flowpath to CB39’s deeper location. Furthermore, the marked correspondence between CB39’s Mg concentration and δ^13^C time series (Fig. 2h) provides tangible evidence that the δ^13^C values were strongly modulated by WRI. High concentrations of dissolved carbonates in CB39’s feeding water, due to more extensive WRIs, caused an elevated SI_CC_, permitting carbonate deposition, and may have been responsible for fabric differences and an extended growth period of CB39 with respect to CB25 and CB47. Such WRIs would mean that a large component of the dissolved inorganic carbon was derived from the host limestone, rather than the soil (as is the case for CB25 and CB47) (e.g.,^20^). Therefore, CB39’s δ^13^C values are not directly related to soil productivity and environmental conditions above the cave. However, the initiation of CB39 formation (Fig. 2) likely corresponded with the onset of thermophilisation, confirmed by the increase in the percentage of woody taxa following Termination II as seen in pollen records from Lago Grande di Monticchio in southern Italy^21^ (Fig. 2d). The following period of stability in woody taxa coincided with the onset of CB47 formation, which was delayed until soil and vegetation above the cave became fully established, creating the conditions required for soil-respiration-instigated speleothem growth.

Speleothem δ^18^O values are often used to constrain the hydrological and environmental conditions during formation, in addition to the moisture source location. The lowest CB39 δ^18^O values are recorded at ~127–126 ka, in analogy to the composite δ^18^O record of Soreq Cave^14^, Israel (Fig. 2b), pointing to a regional hydroclimate response to increasing solar insolation^22^ (Fig. 2a) following Termination II, which also coincided with the onset of CB47 growth (Fig. 2f and g). Sudden decreases in δ^18^O values recorded in speleothems SCH-5 from Schneckenloch Cave in Austria^23^ (Fig. 2c), CC5 from Corchia Cave in Italy^24^ (Fig. 2e) and CB47 (Fig. 2f) at ~125 ka coincided with the onset of CB25 growth, which may have been related to an increase of rainfall, activating CB Cave flowstone development simultaneously to Atlantic storm tracks hitting the western coast of the Italian Peninsula and the Austrian Alps. Increases in CB39 δ^13^C, Mg and Sr records at 124–123 ka (Fig. 2h,i), concomitant with strong fluctuations in δ^18^O values and decreased growth rate of SCH-5 (Fig. 2c)^23^, in addition to cessation of CB47 growth, likely indicate a drying that caused enhanced WRIs in the dripwater feeding CB39. Cessation of CB25 growth at ~121 ka is reasonably explained by a decrease in soil pCO_2_ that resulted in percolation water SI_CC_ inadequate for speleothem formation. The *speleothem limit* and corresponding timberline had, therefore, moved below the altitude of the CB Cave catchment. However, enhanced WRIs of the percolation water feeding CB39 still permitted its formation until the end of the LIG.

**Figure 3:**
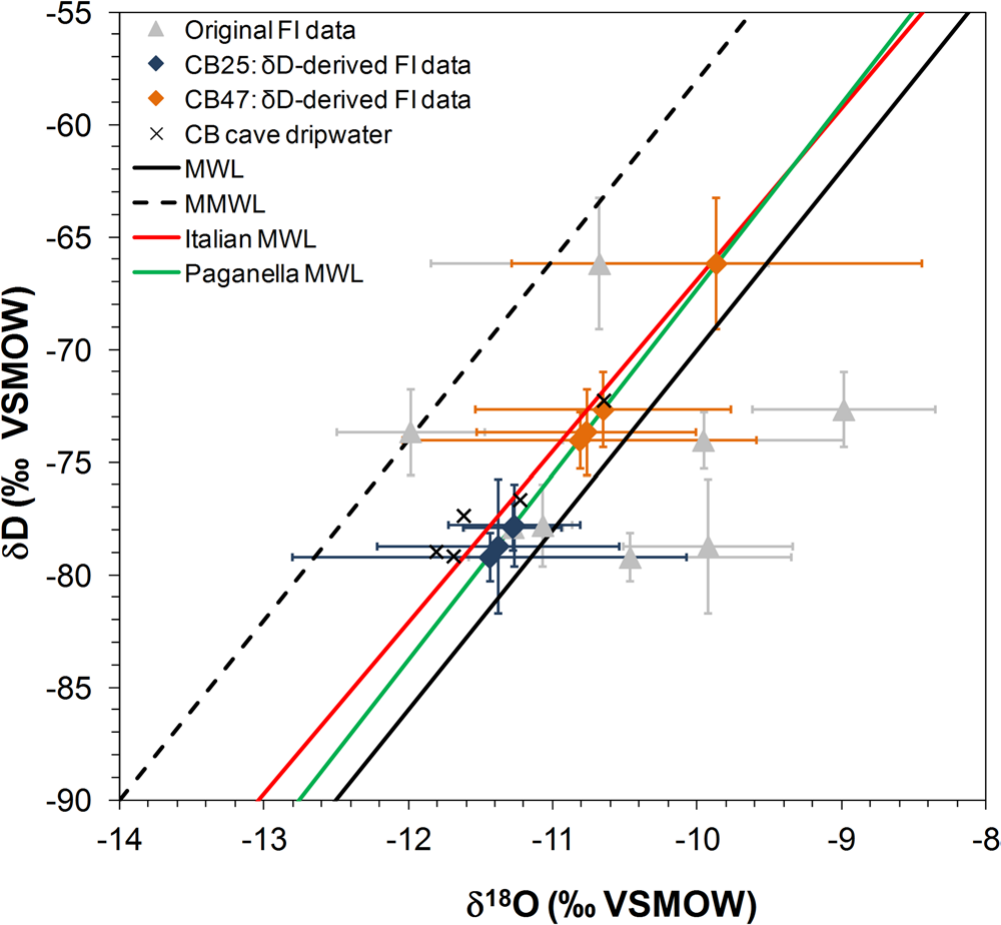
Fluid inclusion data from CB Cave speleothems. The original, measured FI isotopic values (grey) have been adjusted to gain the δD-derived δ^18^O_FI_ values (orange and blue diamonds), calculated from the measured δD_FI_ values, using the modern relationship between δ^18^O and δD at Mt. Paganella. Modern CB Cave dripwaters were taken at 3.4 °C in the Scrigno chamber^11^, which coincides with the 2013–2015 temperature measurements in the chamber (3.45 ± 0.05 °C; Supplementary Fig. S12). Various meteoric water lines (MWL) are also shown: the global MWL (δD = 8·δ^18^O + 10), the East Mediterranean MWL (MMWL; δD = 8·δ^18^O + 22)^50^, the Italian MWL (δD = 7.61·δ^18^O + 9.21)^51^ and the Paganella MWL (δD = 8.24·δ^18^O + 15.1; R^2^ = 0.99) calculated using data from the meteorological station of Mt. Paganella, 2125 m a.s.l.^51^. Raw FI data are found in Supplementary Table S11.

The possibility of LIG thermophilisation at the high-altitude CB Cave site has been tested by constraining the temperatures of the LIG using isotope values from speleothem FIs and their enclosing calcite, using calcite–water oxygen isotope geothermometer equations. Such equations are derived from both experimental^25,26^ and field-based methods^11,27,28^. However, due to inherent disequilibrium isotope fractionation that occurs during speleothem formation, equations characterising near-perfect isotopic equilibrium do not successfully predict speleothem formation temperatures^19,29^. Using cave-derived calcite and water oxygen isotope pairs yields the most accurate speleothem formation temperature reconstructions because the empiric equation includes the intrinsic in-cave fractionation^11,27^. Here, the equation of Johnston *et al*.^11^ was used as the most comprehensive cave-based geothermometer equation available to date (see Supplementary Discussion). The geothermometer equation compares δ^18^O_C_ values with δ^18^O_FI_ values. An unknown in the FI temperature reconstruction is the influence of the transformation of an amorphous carbonate precursor into calcite in a closed system^30^. A fully closed system is unlikely for more porous columnar fabrics where FIs are trapped, however, in the CB Cave samples, this porous fabric was then sealed by compact calcite layers, hampering vertical fluid migration (Supplementary Discussion and Fig. S4). Furthermore, δ^18^O_FI_ values have been shown to change after FI entrapment due to diagenetic processes, including effects of non-classical crystallisation, while the isotope composition of the δD_FI_ values remained unaltered^30^. For this reason, in this study, δ^18^O_FI_ values were calculated using the δD_FI_ values and the modern relationship between δ^18^O and δD in meteoric waters derived from the local meteoric water line constructed using data from Mt. Paganella (Paganella-MWL) (Table 1, Fig. 3). By using the δ^18^O_C_ and the δD-derived δ^18^O_FI_ values as inputs for the geothermometer equation, the resulting speleothem formation temperature estimates are 7.9 ± 1.9 °C for CB47 (126–124 ka) and 3.6 ± 1.5 °C for CB25 (125–122 ka), with the propagation of uncertainties detailed in Supplementary Table S11. When compared with modern Scrigno chamber temperatures (3.45 ± 0.05 °C, Supplementary Fig. S12), the temperature anomalies estimated from FIs are +4.5 ± 1.9 °C at 126–124 ka and +0.2 ± 1.5 °C at 125–122 ka with respect to present-day cave temperatures (Table 2).

**Table 2:** Summary of temperature reconstructions and temperature anomalies.

	CB47		CB25	
	T (°C)^a^	ΔT (°)^b^	T (°C)^a^	ΔT (°C)^b^
FI	7.3 ± 1.9	+ 4.5 ± 1.9	3.0 ± 1.5	0.2 ± 1.5
δ^13^C	7.1 ± 1.3	+ 4.2 ± 1.4	5.5 ± 1.3	+ 2.7 ± 1.4
SI_CC_	7.1 ± 1.4	+ 4.3 ± 1.5	6.8 ± 1.2	+ 4.0 ± 1.3
Mean	7.2 ± 1.6	+ 4.3 ± 1.6	5.1 ± 1.4	+ 2.3 ± 1.4

^a^Mean annual temperature at the infiltration elevation (MAT_inf_).

^b^Temperature anomaly with respect to present-day MAT_inf_.

Temperature estimates derived from FI data were then compared with temperature reconstructions based on geochemical and petrographic data from the CB speleothems. Speleothem geochemistry and calcite fabrics respond to surface air temperature, cave temperature, drip rate and dripwater SI_CC_. By using available datasets from 11 caves in the region^9^ and comparing these with modern and Holocene speleothems from the same caves^11,31^, the temperature and conditions under which geochemical signals are incorporated and fabrics form in the speleothem calcite were reconstructed.

The relationship between infiltration temperature and δ^13^C values for modern speleothems in Trentino can be used to estimate the formation temperatures of LIG sparitic speleothems from their δ^13^C values. Since δ^13^C values of CB39 were strongly affected by WRIs, the calculation was performed for CB25 and CB47 that encode a vegetation signal, which can be related to temperature. To minimise the inclusion of values potentially affected by strong disequilibrium fractionation associated with initiation or cessation of speleothem growth, only the δ^13^C values from the middle sections of the speleothems were used. Using the least fractionated modern speleothem δ^13^C values from Johnston *et al*.^11^ and MAT_inf_ data from Borsato *et al*.^9^, the relationship MAT_inf_ (°C) = −1.68·δ^13^C–7.57 (R^2^ = 0.96) was obtained for sparitic speleothems that formed near isotopic equilibrium (Supplementary Material S14). MAT_inf_ estimates were obtained for the LIG at CB Cave by applying this relationship to the mean of δ^13^C values selected from the least isotopically fractionated middle section of the Scrigno speleothem δ^13^C records. This yielded a mean δ^13^C value of −8.7 ± 0.6‰ (126.0–125.3 ka) for CB47, corresponding to a peak LIG MAT_inf_ of 7.1 ± 1.3 °C and, thus, a peak LIG temperature anomaly of + 4.2 ± 1.4 °C, with respect to modern conditions (Supplementary Material S13). The late LIG, represented by CB25, has a mean δ^13^C value of −7.8 ± 0.7‰ (123.8–121.9 ka), and yielded a late LIG MAT_inf_ of 5.5 ± 1.3 °C, hence an anomaly of + 2.7 ± 1.4 °C, with respect to the modern MAT_inf_ (Table 2).

Alternatively, in a novel approach, we exploit the relationship between SI_CC_ of speleothem-forming water and the coexisting speleothem fabric. Extensive monitoring in the study region provides strong evidence that speleothem fabric is controlled by SI_CC_^7,31^, though a link with the vegetation above the cave, connected with soil and cave air pCO_2_, which is ultimately related to temperature^8,9^. Active columnar fabrics in the region are diagnostic of low carbonate saturation, with SI_CC_ in the range of 0.15–0.35, which also suggests monomer by monomer attachment growth^31^. Whereas, dripwater SI_CC_ for dendritic fabrics is 0.2–0.4, with a mean SI_CC_ that is higher than dripwaters associated with columnar, fibrous and microcrystalline fabrics^7,31^. Therefore, the open and compact columnar fabrics of CB25 likely formed from dripwaters with a low SI_CC_ (0.15–0.35), while mixed columnar–dendritic fabrics of CB47 are expected to have formed under a slightly higher SI_CC_ (0.15–0.4). Although there are uncertainties in the overlap of the field-of-existence of these fabrics, we use this opportunity, where we have various past-temperature estimates, to test the possibility that speleothem fabrics are linked with SI_CC_ and ultimately reflect the temperature and vegetation above the cave. A robust, present-day relationship has been documented between dripwater SI_CC_ and MAT_inf_ in the study region (MAT_inf_ (°C) = 4.36 + 9.95·SI_CC_; R^2^ = 0.93)^9^. While this relationship is robust within our study region, characterised by pure carbonate host rocks in a well-studied climate setting, a lack of research across various settings prevents application to other regions. For CB47, the mean SI_CC_ of 0.28 ± 0.18 corresponds to a peak LIG MAT_inf_ of 7.1 ± 1.4 °C, while for CB25, a mean SI_CC_ of 0.25 ± 0.14 corresponds to a late LIG MAT_inf_ of 6.8 ± 1.2 °C. This suggests an anomaly of + 4.3 ± 1.5 °C at the peak LIG and + 4.0 ± 1.3 °C during the late LIG with respect to present-day MAT_inf_ (Table 2, Supplementary Material S13). The late LIG temperature estimated using the reconstructed-SI_CC_ is slightly higher than the other reconstructions provided here, albeit within the uncertainties, likely reflecting the wide range of possible SI_CC_ values that form columnar calcite. Encouragingly, the peak LIG value gained from the reconstructed-SI_CC_ is remarkably consistent with the temperatures derived from both the FI and δ^13^C methods.

**Figure 4:**
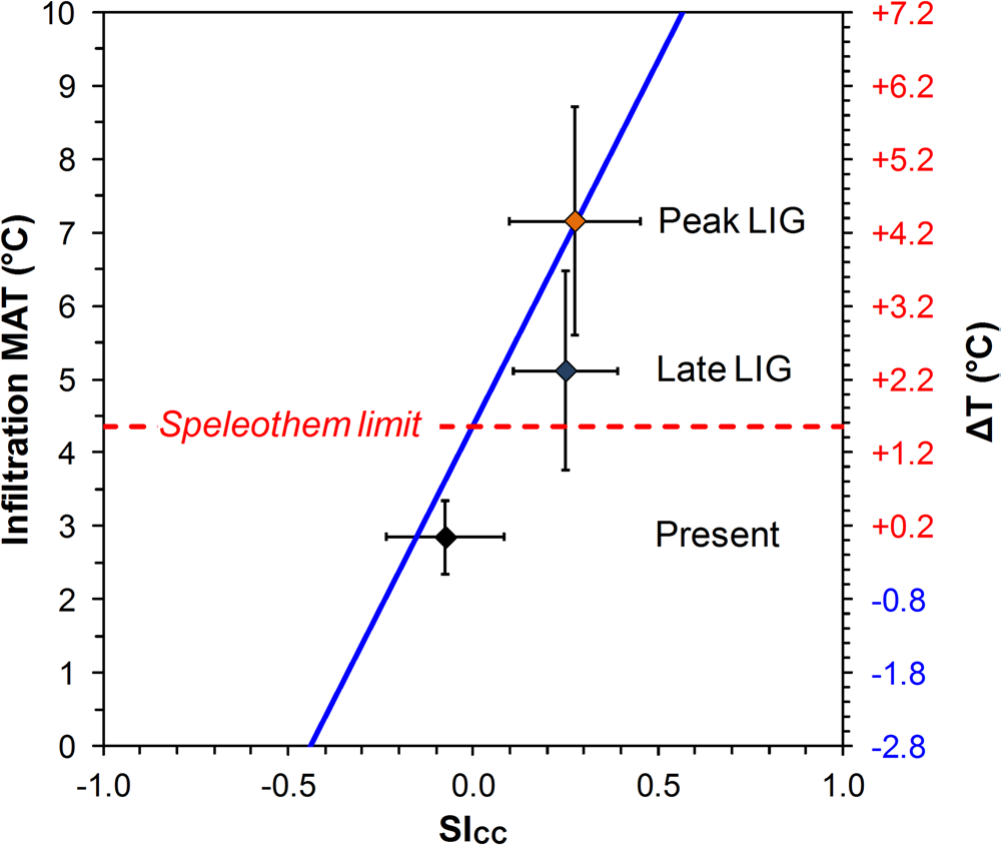
Relationship between dripwater SI_CC_ and the mean annual temperature at the infiltration altitude of the CB Cave catchment area. The blue line represents the regression line between present-day MAT_inf_ (1961–1990) and dripwater SI_CC_ in the region (MAT_inf_ (°C) = 4.36 + 9.95·SI_CC_; R^2^ = 0.93)^9^. Present-day CB Cave dripwater data (2.8 ± 0.5 °C; black diamond) lie below the local *speleothem limit* (at a temperature of 4.4 °C, corresponding to a SI_CC_ = 0, current altitude ~1660 m a.s.l.)^9^ where their negative SI_CC_ values indicate that formation of sparitic speleothem calcite is unlikely. The MAT_inf_ reconstructed for the peak LIG (from CB47) with a SI_CC_ of 0.28 ± 0.18 based on calcite fabrics (orange diamond; 7.2 ± 1.6 °C) lies well above the *speleothem limit*. The late LIG temperature reconstruction (from CB25; blue diamond) with a SI_CC_ of 0.25 ± 0.14 and a MAT_inf_ of 5.1 ± 1.4 °C also lies above the *speleothem limit*.

In summary, temperature anomaly estimates based on different physical and geochemical data from CB speleothems (Table 2) yield a peak LIG temperature anomaly of +4.3 ± 1.6 °C and a late LIG anomaly of +2.3 ± 1.4 °C (+1.4 ± 1.5 °C excluding the reconstructed-SI_CC_ value), with respect to modern temperatures (1961–1990; Fig. 4) at this subalpine site. The good agreement of the independent temperature estimates from three different proxy data grants confidence in our temperature reconstructions and has tested the validity of the fabric–SI_CC_–temperature relationship. The positive temperature anomalies, which are significant with respect to their uncertainties (propagation of uncertainties detailed in Supplementary Table S13), demonstrate that the LIG MAT_inf_ were higher than present-day. In fact, the mere occurence of sparitic speleothems at the altitude of CB Cave already attests to higher-than-present LIG temperatures, and associated better-developed soil and vegetation cover than today.

The magnitude of the peak LIG temperature anomaly calculated here (+4.3 ± 1.6 °C) is within the uncertainty of the +8 ± 4 °C anomaly reconstructed from central Greenland ice cores^32^ and suggests amplification of warming at both high altitudes and latitudes. Over the 4.4 kyrs that encompass the temperature reconstructions from both FIs and δ^13^C values (126.3–121.9 ka) in the CB speleothems, temperature decreased at a rate of 0.5 °C kyr^−1^. This compares with a cooling rate of 0.75 °C kyr^−1^ estimated over the same period of time from temperature reconstructions from Greenland ice cores^32^. In contrast, the global rate of cooling over the same period has been reconstructed at 0.11 °C kyr^−1^ (ref.^33^), implying that, in addition to the magnitude, the rate of temperature change at high altitudes and latitudes was amplified with respect to the global mean.

A LIG temperature increase of +4.3 ± 1.6 °C, associated with thermophilisation, is surmised to have caused an upward shift of the *speleothem limit* to an altitude of ~2500 m a.s.l. and, accordingly, the catchment area of CB Cave at the peak LIG (7.2 ± 1.6 °C) would have been colonised by a mixed deciduous forest typical of the current upper montane zone^8^. This association is now observed in the catchment of Ernesto Cave (ER; Fig. 1), located at 1180 m a.s.l. with a MAT_inf_ of 7.1 °C and a mean dripwater SI_CC_ of 0.22 ± 0.12 (ref.^9^), which contains Holocene stalagmites with columnar, microcrystalline and dendritic fabrics, similar to CB47 (ref.^34^). The temperature of 5.1 ± 1.4 °C calculated from CB25 for the late LIG suggests similar temperatures to those currently experienced in other caves in Trentino, such as “Pozzo di Val del Parol” (VP), located at 1585 m a.s.l. with a MAT_inf_ of 4.8 °C and a dripwater SI_CC_ of 0.12 ± 0.20 (ref.^9^). The VP Cave has active speleothems, including conical stalagmites and drapery stalactites that form below a catchment covered by pastures with scattered conifer trees. Here, the major driving force of modern speleothem development is the vegetation assemblages and associated pCO_2_ of the percolating water that increases the SI_CC_^8^. The increase in LIG temperatures most likely caused significant thermophilisation above CB Cave, thus permitting crystalline speleothems to form.

To test the hypothesis of elevation-dependent warming (EDW) during the LIG, the peak LIG temperature anomaly estimated for the CB site was compared with temperature reconstructions from lower altitude sites. Modelled central European summer temperatures for the LIG maximum are 1–2 °C higher than present^35^. Pollen records in Iberian Margin sediment core MD04–2845 further indicate that the warmest period of the LIG reached temperatures ~2 °C higher than present^36^. Turney and Jones^37^ reconstructed global temperatures 1.5 °C higher than today from a compilation of marine and terrestrial (including ice) proxy records. These temperature anomaly reconstructions are noticeably lower than our peak LIG subalpine speleothem-based estimate of +4.3 ± 1.6 °C. This discrepancy implies that high altitudes were more sensitive to warming than low-altitudes and, thus, argues for EDW during the LIG. Considering the LIG as an analogue for future warming, as expected in a scenario of increased greenhouse gas forcing, applying the low-altitude LIG temperature anomaly data to an alpine region without making adjustments for topography and associated EDW would result in an underestimation of the predicted temperature anomaly. In turn, this would cause an underestimation of the extent of thermophilisation and its impact on the local ecosystems and hydrological cycle, extending to downstream water resources. Using low-altitude temperature estimates to formulate adaptive strategies for mountainous regions is therefore inappropriate. More quantitative temperature reconstructions from well-dated proxy archives at both high and low altitudes in mountainous regions are, therefore, urgently needed.

EDW can be caused by a number of mechanisms including: (i) albedo from snow, ice and vegetation changes, (ii) water vapour and radiative fluxes, (iii) clouds and (iv) aerosols^38^. Our results suggest that the vegetation belts shifted upwards (thermophilisation) at the CB Cave site during the LIG period. This afforestation reduced surface albedo due to increased radiation absorption by plants, enhancing warming in the alpine zone that became more vegetated. As temperatures increased and thermophilisation proceeded, evaporation and evapotranspiration also increased^4^, causing enhanced humidity that has a positive-feedback on warming as water vapour absorbs and emits long-wave radiation. Although a global factor, its non-linear sensitivity means that areas with lower initial humidity, such as cold high-altitude sites, are strongly affected by humidity changes. Therefore, through the positive water vapour feedback, high elevation sites incur enhanced warming relative to areas of high humidity, such as lower altitudes and tropical regions. Today, the temperature gradient (lapse rate) between the valley bottom (200 m a.s.l.; 12.6 °C) and the mountain top (Mt. Paganella, 2125 m a.s.l.; 1.7 °C) is −0.57 °C 100^−1^ m (ref.^39^). If a ~2 °C warming is expected at the valley bottom^35,37^ and +4.3 ± 1.6 °C at 1930 m a.s.l. (present study), the calculated peak LIG altitudinal temperature gradient of −0.4 °C 100^−1^ m implies a reduced rate of cooling with increasing elevation. This suggests that, similarly to observations of current warming at high altitudes^40–42^, EDW likely occurred during the LIG.

Forward-modelling of elevation gradients in the alpine region for the period 2070–2099, against the reference period 1961–1990, indicates a rise of 3.5–5.0 °C for an altitude similar to CB Cave. Winter precipitation is expected to increase in the southern watershed of the Alps while summer precipitation should decrease slightly^43^. Furthermore, maximum changes in albedo are estimated in the altitude band of the CB Cave site with a reduction of 80 snow days per year. This scenario can be compared with the CB Cave reconstruction for the peak LIG with a +4.3 ± 1.6 °C temperature rise. Our data suggest that higher temperatures resulted in high precipitation amounts that provided the water necessary for the formation of flowstones. Data from CB Cave also imply a longer vegetation period, resulting from a reduction in the duration of the snow cover that enhanced greening and soil productivity, which are also beneficial for speleothem formation at high altitudes. The similarity of forward-modelling observations and our LIG reconstruction suggests that by the end of the 21^st^ century, conditions in the southern watershed of the European Alps will likely to be similar to those experienced during the peak of the LIG.

## Methods

### U/Th dating

Fifteen samples from CB25, CB39 and CB47 were analysed for U and Th concentrations at two different laboratories (Supplementary Table S5). At the University of Melbourne, Australia, sub-samples were sawn from visibly clean calcite with vertical dimensions of ~1.5 mm. Chemical separation, measurements of U and Th isotope ratios and multi-collector inductively coupled plasma mass spectrometer (MC-ICP-MS; Nu-instruments Nu Plasma) analysis followed the methods described in Hellstrom^44^. At the University of Minnesota, U.S.A., sub-samples were hand-drilled along growth laminae (width ~1 mm). Chemical and MC-ICP-MS (Finnigan Neptune) analytical methods followed those described in Shen *et al*.^45^. Decay constants of Cheng *et al*.^46^ were used. Corrected ^230^Th ages assume the initial ^230^Th/^232^Th atomic ratio of 4.4 ± 2.2 × 10^−6^, which refer to a material at secular equilibrium with the bulk earth ^232^Th/^238^U value of 3.8. The age datum is defined as 1950 AD and dating uncertainties are reported as ±2 standard errors. The age models of the three speleothems (Supplementary Fig. S6) were developed using the StalAge algorithm^47^, with age uncertainties quoted at the 95% confidence limit.

### Fabric analysis

Uncoated, polished thin sections were observed under plane (PPL) and cross-polarised light (XPL) on a Zeiss Axioskop optical microscope. Fabrics, fabric types, fabric coding and microstratigraphic logging followed the conceptual framework proposed in Frisia^10^, which is based on models of fabric development.

### Stable isotope analysis

Oxygen and carbon stable isotope values were obtained from calcite powders. Flowstones were sampled perpendicular to the visible lamina, whereas stalagmite CB47 was drilled as close as possible to its growth axis but avoiding areas of apparent recrystallization (Supplementary Fig. S3). Micro-milling at increments of 150 µm was used to obtain samples from flowstone CB39, which shows a slow vertical extension rate (4.5 mm kyr^−1^). For CB25 and CB47, whose ages suggest a faster vertical extension rate (18 mm kyr^−1^ and 57 mm kyr^−1^, respectively), sub-samples were obtained by hand-drilling at increments of 1 mm. Stable isotope values of C and O were measured using a Thermo Fisher Delta^Plus^XL mass spectrometer at the University of Innsbruck, Austria. Long-term 2σ reproducibility for δ^13^C and δ^18^O values is ± 0.06‰ and ± 0.08‰, respectively. All results are given in per mil notation with respect to the Vienna Pee Dee belemnite reference material.

### Fluid inclusion analysis

FI isotope data were obtained for CB25 and CB47 only, as the CB39 fabric did not yield sufficient water for analysis. Blocks of calcite ~0.5–1 cm^3^, the minimum volume required to obtain >0.2 μl of water from our samples, were crushed in a custom-built crushing device, in-line with a Delta V Advantage isotope ratio mass spectrometer (IRMS; Thermo Fisher Scientific) at the University of Innsbruck, Austria. Crushed samples were heated at 120 °C in a chamber flushed with helium carrier gas that transported the evolved water vapour to a cryo-focusing cell (−150 °C). The frozen water was subsequently flash-heated to 300 °C and the resulting vapour transferred in a single pulse into the Thermal Combustion/Elemental Analyser (Thermo Fisher Scientific) where it reacted at 1400 °C with glassy carbon, producing H_2_ and CO. The evolved gases were separated in a gas chromatography column and then admitted to the IRMS, where measurements of δD and δ^18^O were carried out^48^. Between each measurement, the line was conditioned with 0.4 μl of in-house reference water. Samples were taken for CB25 at 10–18 mm, 23–27 mm, 38–43 mm and 61–66 mm and for CB47 at 26–42 mm, 53–67 mm, 112–129 mm and 141–152 mm, where distances are calculated from the top of the speleothem (DFT; Supplementary Table S11). Several repeats of each sample were carried out on sub-samples positioned along the same growth layers and the reported uncertainties refer to one standard deviation of these repeat measurements. Results are given in per mil notation relative to Vienna Standard Mean Ocean Water (‰ VSMOW).

### Trace element analysis

Mg/Ca and Sr/Ca ratios were measured at the Research School of Earth Sciences, Australian National University, using laser ablation inductively coupled mass spectrometry^49^. Analyses were carried out as a continuous 33 mm-long transect across the full thickness of sample CB39 and a 36 mm-long transect of the base of the stalagmite CB47. Analyses were conducted with a 200 × 20 µm laser mask image, calibrated with NIST 610 standard and repeated at a lateral offset of 300–400 µm. The repeat track served to identify outliers caused by photomechanical ablation artifacts, which were then removed from the data. Elemental ratios were converted into elemental concentrations using a standard concentration of Ca in calcite of 400,000 ppm (40 wt%). Data were smoothed using a 9-point running average.

## Electronic supplementary material

Supplementary Information

Supplementary Tables S5, S7, S8, S9, S11, S13 and S14
