# Supplementary material for: Evidence of thermophilisation and elevation-dependent warming during the Last Interglacial in the Italian Alps
Source: Sci Rep. 2018 Feb 8;8:2680. doi: 10.1038/s41598-018-21027-3 (PMC5805769; doi:10.1038/s41598-018-21027-3)
Supplement: Supplementary file 1 — Supplementary Information [file 41598_2018_21027_MOESM1_ESM.pdf]

Supplementary Material for:

Evidence of thermophilisation and elevation-dependent warming during the Last Interglacial in the Italian Alps

Johnston, V.E.[a*+], Borsato, A.[a,b], Frisia, S.[b], Spötl, C.[c], Dublyansky, Y.[c], Töchterle, P.[c], Hellstrom, J.C.[d], Bajo, P.[d], Edwards, R.L.[e] and Cheng, H.[e,f].

[a]Museo delle Scienze, Corso del Lavoro e della Scienza, 3, 38122 Trento, Italy (*correspondence: johnston.ve@gmail.com)
[b]School of Environmental and Life Sciences, University of Newcastle, Callaghan, 2308 NSW, Australia
[c]Institute of Geology, University of Innsbruck, Innrain 52, 6020 Innsbruck, Austria
[d]School of Earth Sciences, University of Melbourne, Melbourne, 3010 VIC, Australia
[e]Department of Earth Sciences, University of Minnesota, Minneapolis, USA
[f]Institute of Global Environmental Change, Xi'an Jiaotong University, Xi'an, China
[+]Current address: Karst Research Institute, Research Centre of the Slovenian Academy of Sciences and Arts, Titov trg 2, SI-6230 Postojna, Slovenia.

Contents

Supplementary Fig. S1. Cross-section of the geological setting surrounding the research site.

Supplementary Fig. S2. Simplified vertical cross section of CB cave.

Supplementary Fig. S3. Images of the studied speleothem samples.

Supplementary Fig. S4. Photomicrographs and SEM images of selected fabrics in flowstone CB25.

Supplementary Discussion. Fabric descriptions.

Supplementary Table S5. U/Th activities and age determination.

Supplementary Fig. S6. Age models of the three Cesare Battisti cave speleothems.

Supplementary Table S7. Isotopic time-series data for flowstone CB25.

Supplementary Table S8. Isotopic time-series data and petrography for flowstone CB39.

Supplementary Table S9. Isotopic time-series data for stalagmite CB47.

Supplementary Fig. S10. $\delta^{18}O$ vs. $\delta^{13}C$ values for speleothems.

Supplementary Table S11. Fluid inclusion isotope raw data.

Supplementary Discussion. Fluid Inclusion robustness.

Supplementary Discussion. Geothermometry.

Supplementary Fig. S12. Measured temperature in the Scrigno chamber.

Supplementary Material S13. Calculations for alternative temperature estimates.

Supplementary Material S14. Modern temperature gradients versus $\delta^{13}C$ values in Trentino.

Supplementary References.

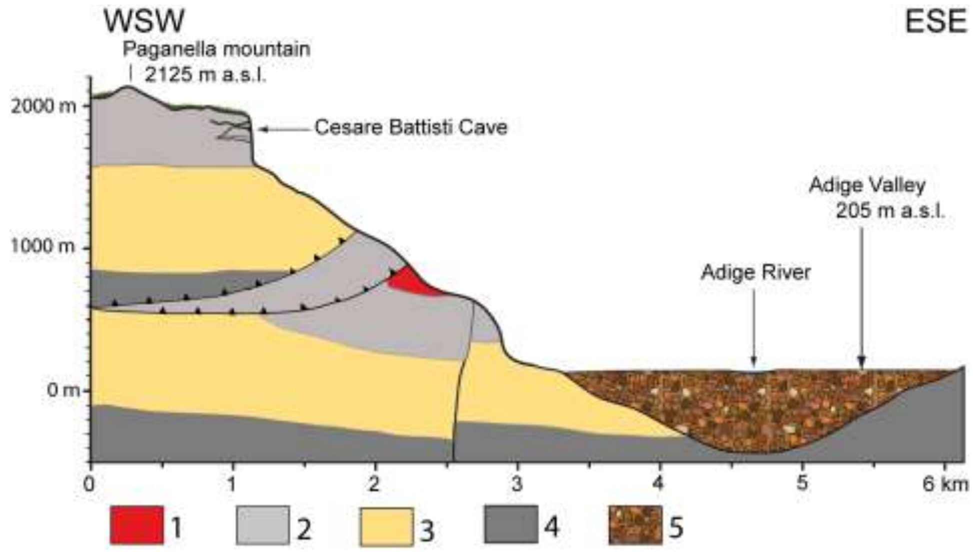

Supplementary Fig. S1. Cross-section of the geological setting surrounding the research site. The highest point is Mt. Paganella (2125 m a.s.l.) and the Adige Valley forms the lowest surface (205 m a.s.l.). Rock formations are: 1) Cretaceous to Middle Jurassic nodular marly limestones, 2) Lower Jurassic limestones (*Calcari Grigi*; CB cave host-rock), 3) Upper Triassic dolomites (*Dolomia Principale*), 4) Middle to Lower Triassic sedimentary sequence and 5) Quaternary deposits. During the last glacial maximum, ice filled the Adige valley to a level just below the cave entrance. CB cave developed in Calcari Grigi; a typical shallow-marine carbonate platform facies ($\delta^{13}C$ = +1‰ to +3‰ and $\delta^{18}O$ = −6 to −2‰) (Borsato et al., 1994).

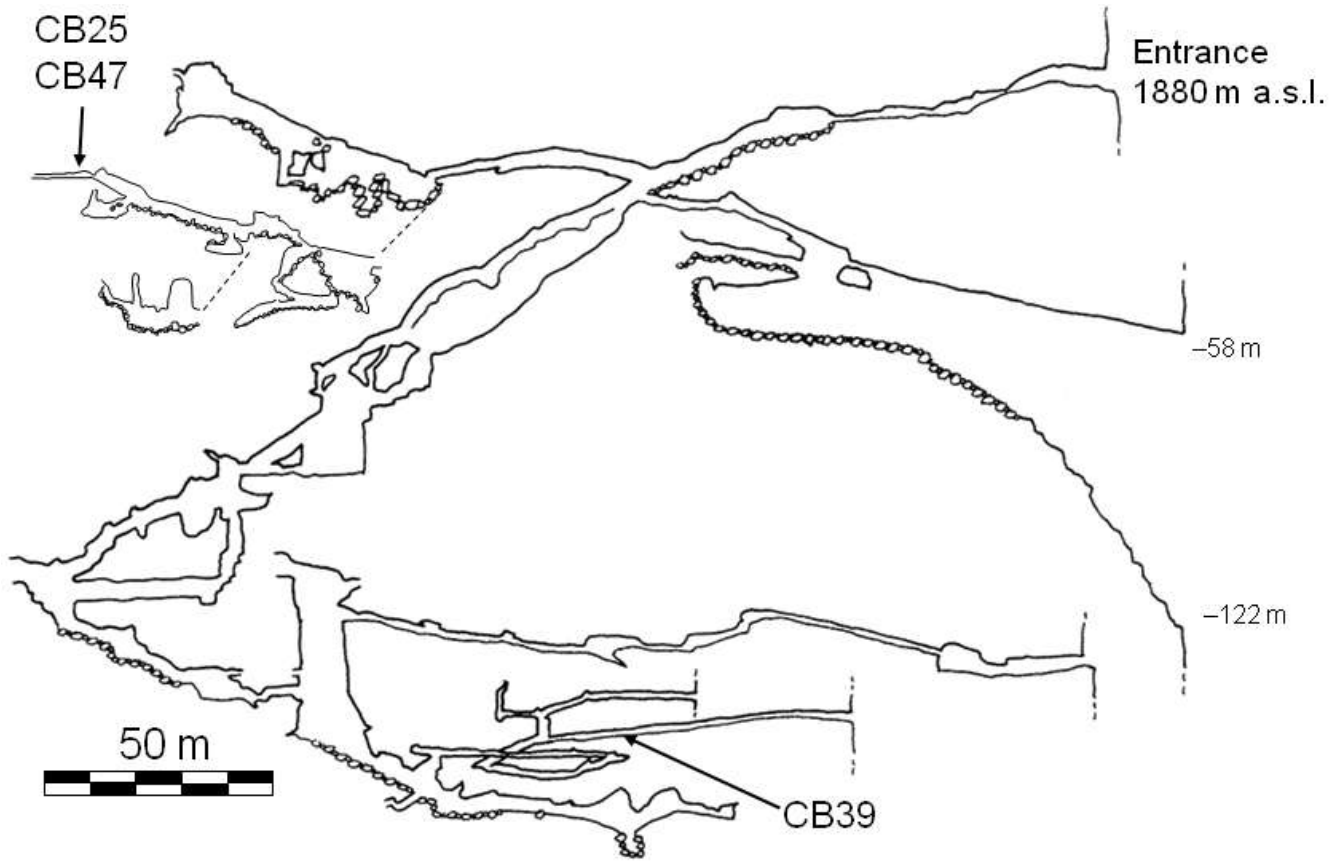

Supplementary Fig. S2. Simplified vertical cross-section of CB cave. The sampling points are marked by arrows. Note that the numerous passageways, shown here on the right-hand side, open onto the cliff wall (illustrated with vertical dashed lines at the end of the passageways). CB25 and CB47 were recovered from the small chamber called the *Scrigno*, located ~70 m below the present-day surface (1930 m a.s.l.). The CB39 sample was taken from a deeper passageway developed ~130 m below the present-day surface and relatively close to one of the many cave entrances on the cliff wall.

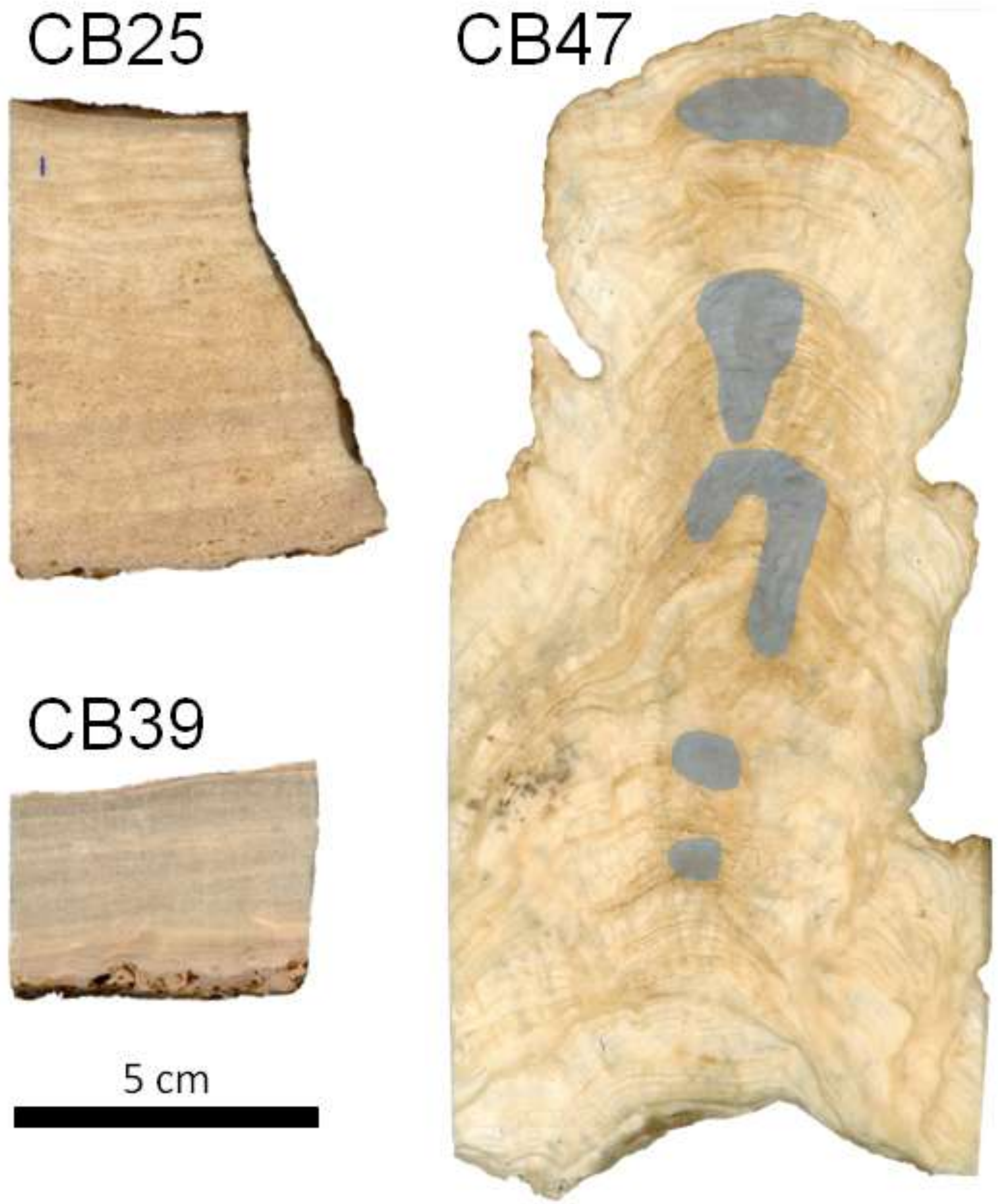

Supplementary Fig. S3. Polished slabs of the studied speleothem samples. Sampling of the speleothems was carried out to minimise the impact on the cave environment, by utilising naturally broken speleothems and small blocks removed from larger flowstone slabs. CB47 shows zones of slight recrystallisation, mostly confined to the central parts (approximately marked as semi-transparent blue shading), which were avoided—as best as possible—when sampling for ages used in the age model construction and for stable isotopes.

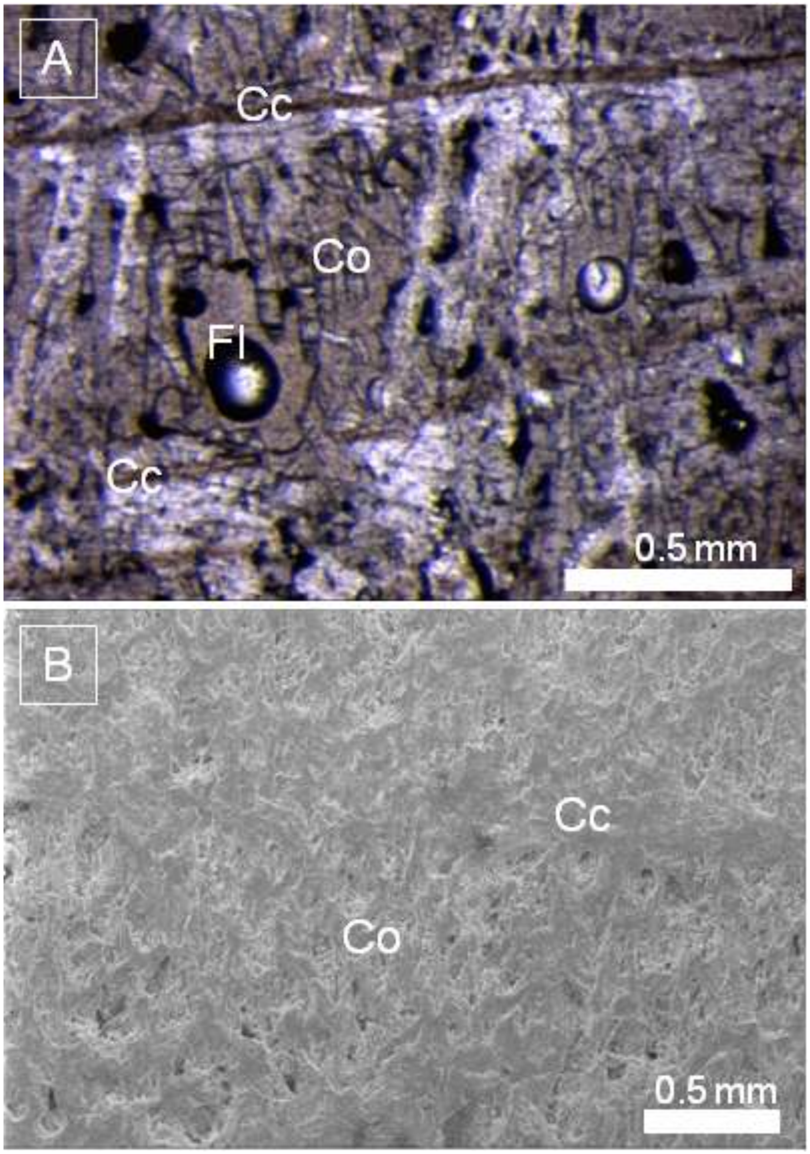

Supplementary Fig. S4. Photomicrograph (a) and Scanning Electron Microscope (SEM) image (b) of CB25 showing the alternating compact columnar (Cc) and porous, open columnar (Co) fabrics. The alternating layers trap fluid inclusions (FI) in their porous portions, while the successive compact portions prohibited interconnecting porosity on a scale larger than the layering, which may correspond with environmental cycles, such as seasonal or annual growth, and shows that the fluid inclusion analyses were performed on coeval calcite–water sample pairs.

Supplementary Discussion: Fabric descriptions.

Flowstone CB25 consists of stacked, lens-shaped layers of milky, open columnar (Co) calcite characterised by high intercrystalline porosity, separated by mm-thin layers of compact columnar (Cc) calcite, which also compose its top 5 mm and a layer at ~50–55 mm (DFT). Stalagmite CB47 mostly consists of milky Co fabrics with high intercrystalline porosity at 40–140 mm. Micrite (M) interrupts Co at ~90 mm. The upper 35 mm of CB47 consists of dendritic fabric (D) and the transition to the porous Co occurs over ~10 mm of columnar microcrystalline (Cm) fabric. Flowstone CB39 consists mainly of elongated columnar calcite (Ce; Supplementary Material S3), with M layers at ~1.1, 1.5, 26, 30 and 31 mm (DFT), corresponding to ~120.6, 120.7, 126.3, 127.0 and 127.3 ka, respectively, and elongated columnar calcite with lateral overgrowths (Ce_{lo}) at 3–6, 10–14, 22–26 and 31–33 mm, corresponding to ~121, 123, 126 and 127 ka, respectively (see Fig. 2 in main text).

Both CB25 and CB47 are dominated by highly porous Co fabric, suggesting that most of their growth history was influenced by relatively high, irregular drip rates (Frisia, 2015). CB47 terminates with a D fabric. The D fabric commonly develops when there is variable seasonal drip rates and episodic high growth rates. CB25 terminates with a Cc fabric. In a flowstone characterised by high intercrystalline porosity, the Cc fabric likely indicates diminished discharge compared with its previous phase of formation (Frisia, 2015). The presence of M in CB47 suggests a period of low flow.

CB39 is characterised by the lack of intercrystalline porosity, possibly related to a steady carbonate load derived from water-rock interactions. However, the presence of layers of micrite and calcite with lateral overgrowths suggests periods of increased discharge and the presence of impurities (Frisia, 2015). The top of the flowstone is marked by M and a thin layer of Ce capped by a thin calcified

detrital layer. In similarity with the M layers in the Scrigno speleothems, it is reasonable to infer that discharge diminished rather abruptly (M layer), then briefly recommenced, to stop after ~100–200 years.

Supplementary Table S5. U/Th activities and age determination. Please see spreadsheet.

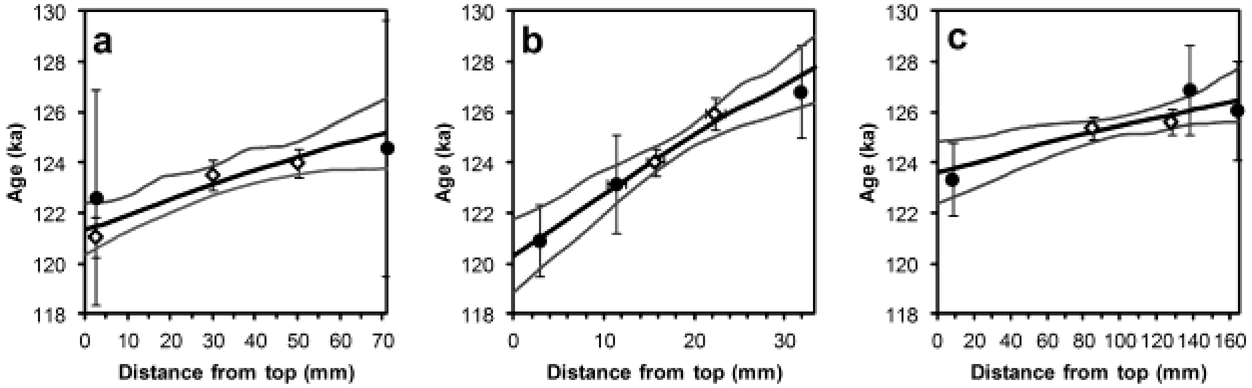

Supplementary Fig. S6. Age models of the three CB cave speleothems. a) CB25, b) CB39 and c) CB47 are plotted with the same age (y-axis) scale. All age models (thick black line) and their associated uncertainties (thinner grey lines) were produced using StalAge (Scholz and Hoffman, 2011). Data are from the laboratories at the Universities of Melbourne (M; filled circles) and Minnesota (MN; open diamonds).

Supplementary Table S7. Isotopic time-series data for flowstone CB25. Please see spreadsheet.

Supplementary Table S8. Isotopic time-series data and petrography for flowstone CB39. Please see spreadsheet.

Supplementary Table S9. Isotopic time-series data for stalagmite CB47. Please see spreadsheet.

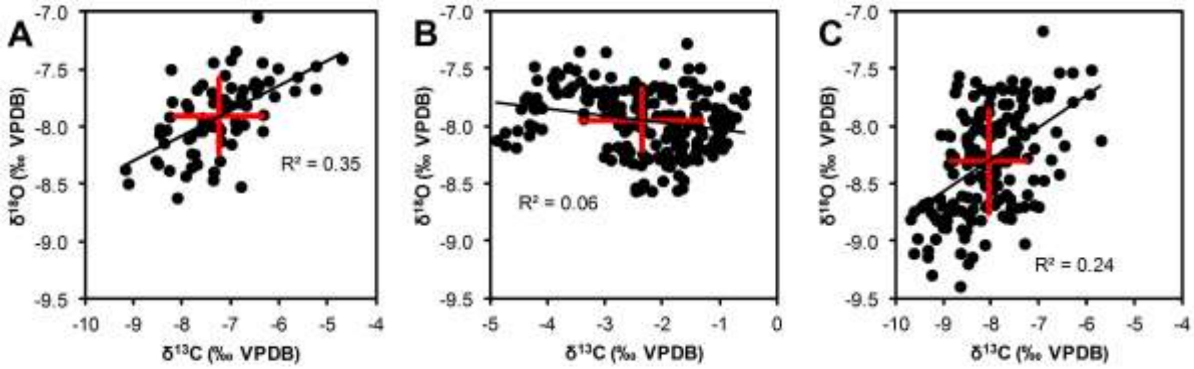

Supplementary Fig. S10. $\delta^{18}O$ vs. $\delta^{13}C$ values for speleothems A) CB25, B) CB39 and C) CB47 as a possible indicator of isotope disequilibrium effects. The red crosses indicate the mean value and standard deviation of the data. Note that the x-axis of B is on a different scale to the other panels. Isotope data are found in Supplementary Tables S7–S9 (see spreadsheet).

Supplementary Table S11. Fluid inclusion isotope raw data. Please see spreadsheet.

Supplementary Discussion: Fluid inclusion robustness

Given that CB25 has a porous fabric, there was the possibility that FIs were not coeval with the surrounding calcite, as more recent fluids could have percolated through interconnected pores. To test the interconnectivity of pore spaces, two additional blocks from the same growth layers as CB25-B were submerged in two isotopically different standard solutions for 24 hours. A significant difference in the measured isotopic composition of the FIs in these soaked samples in comparison with those from the original (un-soaked) sample would indicate interconnected pores and possible contamination from more recent percolation water. By contrast, if the soaked samples had similar FI results to the un-soaked sample, this would indicate that the FIs likely formed contemporaneously with the surrounding calcite and, therefore, the formation temperature can be reconstructed using the relationship between $\delta^{18}O$ values of the FI-derived water ($\delta^{18}O_{FI}$) with that of the coeval calcite ($\delta^{18}O_{C}$).

We tested the possibility of fluid exchange through interconnected pores by soaking CB25-B sample (CB25-B: $\delta^{18}O_{FI}$ = −11.0 ± 0.5‰, δD_{FI} = −78.8 ± 1.7‰) in standard solutions Haus1 ($\delta^{18}O$ = −1.4‰, δD = −12.3‰,) and Haus2 ($\delta^{18}O$ = −29.2‰, δD = −230‰). When soaked in Haus1, the sub-sample (CB25-H1: $\delta^{18}O_{FI}$ = −11.6‰, δD_{FI} = −74.5‰) showed little change compared with the same, un-soaked sample. Furthermore, when soaked in Haus2, the sub-sample (CB25-H2: $\delta^{18}O_{FI}$ = −10.6‰, δD_{FI} = −86.5‰) yielded similar values to those of the un-soaked sample (CB25-B).

The fabrics of CB25 and CB47 are characterised by alternating compact and porous layers (see Supplementary discussion: fabric descriptions). FIs were trapped in the porous Co layers. These Co layers are thought to have formed with a high vertical extension rate under a relatively fast discharge rate, which suggests that the porous Co layers containing the FIs were influenced by relatively little disequilibrium fractionation (Deininger et al., 2012). The more compact C laminae then sealed each porous layer, thus entrapping the fluid inclusion water and confining it to a single layer that may have formed under an environmental influence, such as seasonal or annual layering. A lower discharge rate forming the compact C laminae may have promoted isotope disequilibrium only in these very thin laminae, which would not have a significant effect on the $\delta^{18}O_C$ values. This may explain how the $\delta^{18}O_{FI}$ and δD_{FI} values of the sub-samples soaked in standard solutions with significantly different isotopic composition yielded similar values to the un-soaked CB25 sample. We can conclude that the CB25 fabric does not have interconnected pores across laminae, preventing vertical fluid flow, and thus assume that FI water is coeval to the enclosing calcite. Conversely, FIs could also be produced by infiltration water flowing through an open fabric, followed by the healing of pores and fractures by precipitation of secondary calcite. However, we do not find evidence for this process in CB25, which appears to retain an original texture of alternating Cc and Co fabrics. Some alteration has been

identified in areas of CB47 and the origin of these FIs is therefore questionable. However, the zones selected for FI analyses in CB47 showed negligible alteration, while the excellent correspondence between the temperatures gained from the FIs and the temperature reconstructions gained from $\delta^{13}C$ values and fabric reconstructed-SI_{CC} boosts our confidence in the results and highlights the need for multiple, independent temperature estimates when deducing past temperatures from speleothems.

Supplementary Discussion: Geothermometry.

There is currently much debate over which of the calcite–water oxygen isotope fractionation equations most accurately represent equilibrium conditions. This is particularly important in the case of speleothems that are known not to form at equilibrium, with disputes over which of these should be used for calculating temperatures for speleothem formation. Experimentally derived equations include minimal to negligible disequilibrium isotope fractionation. However, speleothems are known to be affected by in-cave fractionation, and therefore, the temperatures estimated using the experimentally derived equations do not reflect accurately the natural cave environment.

A number of methods have been proposed to overcome this issue in speleothems, including combining FI analysis with clumped isotope analysis (see Daëron et al., 2011; Wainer et al., 2011), making a correction based on a fixed offset calculated from differences between temperatures measured in the cave and those calculated from FIs in modern calcite (Matthews et al., 2000), or using empirical equations calculated from modern cave calcite–water pairs (Tremaine et al., 2011; Johnston et al., 2013). Since there is not adequate calcite for FI analysis currently forming in CB cave for a correction based on a fixed temperature offset, our only option is to use the empirical equations calculated from modern cave calcite–water pairs.

There are a number of different equations derived from field measurements. The equation derived by Coplen (2007) was based on measurements of a Devil's Hole calcite vein that formed below the water table at a temperature of 33.7°C; the equation ($1000\ln \alpha_{calcite–water} = 17.4 (1000/T) - 28.6$) was deemed satisfactory between 13°C and 40°C, which is above the limit of possible temperature estimates for CB cave. Due to the slow, subaqueous formation of this calcite vein, this equation supposedly reflects thermodynamic equilibrium and, thus, does not include the in-cave disequilibrium isotope fractionation that is required here to gain a realistic cave temperature and is, therefore, excluded from our temperature estimate. The equation derived by Tremaine et al. (2011) ($1000\ln \alpha_{calcite–water} = 16.1(1000/T) - 24.6$) included data from their own study (measured between 12°C and 21.5°C) plus various cave calcite data from the literature (between 1.8°C and 26.6°C). This means that the equation derived by Tremaine et al. (2011) would be a good candidate for the calculation of temperature as it intrinsically includes in-cave disequilibrium isotope fractionation and covers the range of temperature values expected for CB cave. An improvement in this equation was subsequently made by Johnston et al. (2013) who used an increased bank of data from the literature (including all those in Tremaine et al. (2011)), in addition to their own measurements that focused on the low temperature range of that typically found in the caves of Trentino and included data from CB cave itself ($1000\ln \alpha_{calcite–water} = 17.66(1000/T) – 30.16$). Therefore, we have selected the equation of Johnston et al. (2013) to use here for geothermometry calculations from calcite–water oxygen isotope data from CB cave speleothems.

There are limitations with respect to the technique of calculating temperatures from calcite–water oxygen isotope data, especially in speleothems with an open texture that formed in cold climates—such as CB25 and CB47—that are susceptible to diagenetic alteration (Demény et al., 2016). Neither the δD_{FI} nor the $\delta^{18}O_{FI}$ values, however, show a correspondence with the distance from the top of the

speleothems, hence, if alteration were present, there would be an insignificant correlation between alteration and time elapsed since deposition. Nevertheless, the $\delta^{18}O_{FI}$ values used for the geothermometry calculations were re-evaluated based on the δD_{FI} values and the modern relationship between $\delta^{18}O$ and δD in meteoric waters derived from the local meteoric water line constructed using data from Mt. Paganella. Accordingly, we eliminate the effect of any post-depositional diagenetic alteration on the $\delta^{18}O_{FI}$ values by using the δD_{FI} values that have been showed to be robust, even in speleothems that have undergone significant diagenetic alteration (Demény et al., 2016).

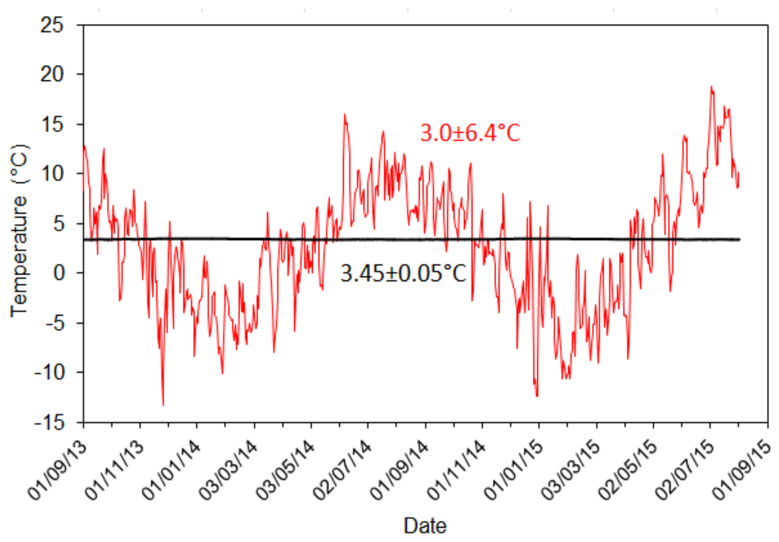

Supplementary Fig. S12. Hourly measured temperature in the Scrigno chamber; September 2013–August 2015 (black line, average: 3.45 ± 0.05°C) compared with the mean daily surface air temperatures measured at Mt. Paganella (2125 m a.s.l.) meteorological station (red line, average: 3.0 ± 6.4°C: www.meteotrentino.it).

Supplementary Material S13. Calculations for alternative temperature estimates. Please see spreadsheet.

Supplementary Material S14. Modern temperature gradients versus $\delta^{13}C$ values in Trentino. Please see spreadsheet.

Supplementary References

Borsato A., Frisia S. and Sartorio D. (1994) Late Triassic - Early Jurassic evolution at the margin between the Trento Platform and the Lombardy Basin (Brenta Dolomites, Italy). *Studi Trentini di Scienze Naturali. Acta Geologia* **69**, 5–35.

Coplen T. B. (2007) Calibration of the calcite-water oxygen-isotope geothermometer at Devils Hole, Nevada, a natural laboratory. *Geochim. Cosmochim. Acta* **71**, 3948–3957. doi:10.1016/j.gca.2007.05.028.

Daëron M., Guo W., Eiler J., Genty D., Blamart D., Boch R., Drysdale R., Maire R., Wainer K. and Zanchetta G. (2011) $^{13}C^{18}O$ clumping in speleothems: Observations from natural caves and precipitation experiments. *Geochim. Cosmochim. Acta* **75**, 3303–3317. doi:10.1016/j.gca.2010.10.032.

Deininger M., Fohlmeister J., Scholz D. and Mangini A. (2012) Isotope disequilibrium effects: The influence of evaporation and ventilation effects on the carbon and oxygen isotope composition of speleothems - A model approach. *Geochim. Cosmochim. Acta* **96**, 57–79. doi: 10.1016/j.gca.2012.08.013.

Demény A., Czuppon G., Kern Z., Leél-Össy S., Németh A., Szabó M., Tóth M., Wu C.-C., Shen C.-C., Molnár M., Németh T., Németh P. and Óvári M. (2016) Recrystallization-induced oxygen isotope changes in inclusion-hosted water of speleothems – Paleoclimatological implications. *Quatern. Int.* **415**, 25–32. doi:10.1016/j.quaint.2015.11.137.

Frisia S. (2015) Microstratigraphic logging of calcite fabrics in speleothems as tool for palaeoclimate studies. *Int. J. Speleol.* **44**, 1–16.

Johnston V. E., Borsato A., Spötl C., Frisia S. and Miorandi R. (2013) Stable isotopes in caves over altitudinal gradients: fractionation behaviour and inferences for speleothem sensitivity to climate change. *Clim. Past.* **9**, 99–118. doi:10.5194/cp-9-99-2013.

Matthews A., Ayalon A. and Bar-Matthews M. (2000) D/H ratios of fluid inclusions of Soreq cave (Israel) speleothems as a guide to the Eastern Mediterranean Meteoric Line relationships in the last 120 ky. *Chem. Geol.* **166**, 183–191. doi:10.1016/S0009-2541(99)00192-8.

Scholz D. and Hoffman D. L. (2011) StalAge - an algorithm designed for construction of speleothem age models. *Quat. Geochronol.* **6**, 369–382. doi:10.1016/j.quageo.2011.02.002.

Tremaine D. M., Froelich P. N. and Wang Y. (2011) Speleothem calcite farmed in situ: Modern calibration of $d^{18}O$ and $d^{13}C$ paleoclimate proxies in a continuously-monitored natural cave system. *Geochim. Cosmochim. Acta* **75**, 4929–4950. doi: 10.1016/j.gca.2011.06.005.

Wainer K., Genty D., Blamart D., Daëron M., Bar-Matthews M., Vonhof H. B., Dublyansky Y., Pons-Branchu E., Thomas L., van Calsteren P., Quinif Y. and Caillion N. (2011) Speleothem record of the last 180 ka in Villars cave (SW France): Investigation of a large $d^{18}O$ shift between MIS6 and MIS5. *Quat. Sci. Rev.* **30**, 130–146. doi:10.1016/j.quascirev.2010.07.004.
